# Mechanical stability of the interaction between a lower-limb rehabilitation exoskeleton and the user’s body during functional tasks

**DOI:** 10.3389/fbioe.2026.1733173

**Published:** 2026-03-20

**Authors:** Simona Squartecchia, Marco Rabuffetti, Tiziana Lencioni, Johanna Jonsdottir, Alberto Marzegan, Pietro Di Bello, Indya Ceroni, Marianna Semprini, Maurizio Ferrarin

**Affiliations:** 1 IRCCS Fondazione Don Carlo Gnocchi, Milan, Italy; 2 Istituto Italiano di Tecnologia, Genoa, Italy; 3 Department of Informatics, Bioengineering, Robotics and Systems Engineering (DIBRIS), University of Genoa, Genoa, Italy

**Keywords:** ergonomics, exoskeleton, kinematic discrepancy, locomotion, mechanical stability, wearable robotics

## Abstract

**Introduction:**

Lower-limb exoskeletons support gait restoration in individuals with locomotor impairments. Mechanical stability in human-exoskeleton interaction is essential for safety. This pilot study evaluated the interaction stability between TWIN exoskeleton and user by analyzing relative displacements between the exoskeleton cuffs and the corresponding user’s thigh and shank during walking (WA) and sit-to-stand (STS). A second parameter quantified the discrepancy between exoskeleton and user joint angles.

**Methods:**

Five healthy adults performed STS and WA while wearing TWIN. An optoelectronic system tracked markers placed on anatomical landmarks and exoskeleton segments. The standard deviation of the relative displacement (RDstd) between the exoskeleton and the underlying anatomical segment quantified mechanical stability. Human-exoskeleton kinematic discrepancy (HEKD) was determined as root mean square of the difference between encoder- and motion capture-derived joint angles.

**Results:**

During STS, RDstd was 4.6 ± 1.5 mm (thigh) and 1.4 ± 0.1 mm (shank). During WA, values were 3.3 ± 0.4 mm (thigh) and 2.7 ± 0.2 mm (shank). HEKD during WA was 1.7 ± 0.6 deg (hip) and 1.9 ± 0.4 deg (knee), whereas 2.2 ± 0.8 deg (hip) and 3.3 ± 1.3 deg (knee) during STS. No participants reported discomfort or pain during tests.

**Discussion:**

All relative displacement values were below 11.7 mm (safety value for the possible onset of skin damages). Larger relative displacements at thigh level during STS than during WA were likely due to larger hip and knee joint excursions associated to STS. Conversely, larger relative displacements at shank level during WA than during STS can be attributed to foot impact forces occurring during WA. Compared to the results from a previous study on a different Hip Active Orthosis, which reported RDstd values at the thigh between 4.0 and 6.3 mm during walking, TWIN showed smaller values, confirming a greater mechanical stability at that level. The small values of discrepancy between exoskeleton and anatomical joint angles during both tasks (below 2 deg during WA and 3.3 deg during STS) indicated strong human-exoskeleton kinematic coupling. Taken together, these findings suggest that TWIN provided high stability during both motor tasks without compromising comfort or safety.

## 1 Introduction

In recent years, exoskeletons have gained increasing attention in various fields, including biomedical research and clinical rehabilitation (Bogue, 2009; Pons, 2010). A particularly significant area of application concerns gait rehabilitation, especially for people with neurological or musculoskeletal impairments. Lower-limb exoskeletons are primarily designed to facilitate the restoration of a physiological gait pattern by providing body stability and leg movement support throughout the walking cycle. The use of active orthoses, such as powered exoskeletons, has demonstrated promising outcomes in improving mobility and promoting functional independence in individuals with compromised lower limb function. Importantly, their therapeutic efficacy strongly depends on the user’s level of active participation, as such engagement fosters motor recovery and neuroplasticity, especially in individuals with residual motor function (Fisher and Sullivan, 2001; Gao et al., 2026; Krakauer, 2006).

Active exoskeletons are often recommended for patients affected by spinal cord injury (SCI) or post-stroke motor deficits. In case of complete lower limb paralysis, for example in complete SCI, position control mode is typically employed, which imposes predefined trajectories to each actuated joint, in order to mimic physiological movements. Conversely, when residual motor function is still present, for example in incomplete SCI or partial hemiparesis following a stroke, assist-as-needed control mode is employed, in order to provide assistance that is adaptable and proportional to the user’s residual motor capabilities, by tuning the specific parameters according to both the feedback from the subjects and visual observation of the gait pattern (de Miguel-Fernández et al., 2023). This personalized approach encourages active involvement and avoids passive motor execution. Evidence suggests that greater frequency, duration and consistency of exoskeleton use during rehabilitation correlates with improved patient outcomes (Nepomuceno et al., 2024).

A critical factor influencing the performance and safety of powered exoskeletons is the quality of the human–robot interface. In particular, the design of the physical interface, commonly consisting of fabric cuffs, is pivotal, as it directly affects ergonomics, comfort, and usability (Cempini et al., 2012). An important factor such as comfort has to be taken into account when developing an exoskeleton, as long as biomechanical and functional requirements are assured (Laffranchi et al., 2021). It is important to ensure that the exoskeleton provides the performance for which it is required and designed, while also preventing overloading of the joints, as well as injuries and abrasions resulting from prolonged use of the device during rehabilitation sessions. Therefore, both comfort and efficiency must be guaranteed. Excessive pressures or shear forces at the contact points between the exoskeleton and the user’s body are often due to joint axis misalignment, leading to sliding movement along the human limb, which may result in inconsistent assistive torque transmission, pain and potential skin wounds (Schiele, 2008; Schiele and Van Der Helm, 2006), while also reducing comfort and safety (Naf et al., 2018; Rocon et al., 2008). This represents a well-known safety issue in rehabilitation robotics (Bessler et al., 2020; 2021) occurring when the exoskeleton joints are not correctly aligned with the corresponding anatomical ones. Misalignment can result from incorrect positioning or fitting of the device (as the exoskeleton joint axis can be shifted with respect to the human joint axis), or from a kinematic mismatch between the two joints (often due to the geometry of human articulations which are biomechanically complex and cannot be perfectly replicated by the exoskeleton joints (Mallat et al., 2019). Therefore, an adequate human-robot joint axis alignment is not easy to achieve. Only in recent years joint misalignment and human–exoskeleton mechanical interaction has emerged as an important topic in the design and evaluation of wearable robotic devices. Despite growing interest, there is currently no consensus on standardized methods to quantify misalignment, as highlighted by (Massardi et al., 2022). Existing approaches predominantly rely on the direct measurement of interaction forces. In this work, we adopt a kinematics-based approach to assess mechanical stability and joint alignment during exoskeleton use. While joint kinematics has been explored in a limited number of studies (Massardi et al., 2022), it remains underutilized compared to force-based methods, despite its practicality and broad applicability to devices not equipped with force sensors.

Discomfort issues could escalate if the materials adopted for the cuffs are inadequate or if the design of these parts is suboptimal (Massardi et al., 2023a). For these reasons, the cuffs must ensure a secure and comfortable connection with the subject, enabling a safe and stable walk. Even though physical interaction has not been deeply investigated (Massardi et al., 2022; Massardi et al., 2023a), it has been found that the majority of adverse events during the use of exoskeletons for gait rehabilitation are associated to human-device interaction (Bessler et al., 2020).

One of the devices designed for the rehabilitation of individuals with neurological and muscular impairments is TWIN, a powered lower-limb exoskeleton developed at IIT-INAIL Rehab Technologies Lab (Laffranchi et al., 2021). TWIN can provide complete assistance by position control of predefined trajectories, or adaptive support through the TWIN-Acta control suite (Semprini et al., 2022). The key feature of the system TWIN equipped with TWIN-Acta is its ability to support the re-establishment of a physiological gait pattern based on the user’s residual motor skills, rather than forcing a predefined trajectory. Originally designed for patients with complete SCI, who need lower limb bilateral complete assistance, TWIN has been adapted to address the needs of post-stroke users as well, i.e. assist-as-needed unilateral support for the hemiplegic leg. The mechanical structure of TWIN is composed of a pelvis module and two lower limbs, each actuated by two active joints located at the hip and knee. The length of the thigh (hip-knee distance) and of the shank (knee-ankle distance) can be adjusted to fit the user anthropometry. Assistance is provided in a task-specific and demand-driven manner, rather than continuously, allowing for interaction that is responsive to the user’s motor abilities. One of the main aspects of this system is the user-exoskeleton interface, provided by specific exoskeleton fabric cuffs that must be fastened around the corresponding body parts (thigh or shank). These cuffs represent a critical design feature, as they must ensure maximum comfort while guaranteeing the stability and walking performance required. In this context, ergonomics plays a fundamental role in ensuring that the device adapts properly to the user’s body, minimizing discomfort, pressure points and the risk of skin injuries during prolonged use.

In the present pilot study, an experimental trial was conducted on five healthy volunteers that performed several tasks – including sit-to-stand (STS) and walking (WA) – while wearing the TWIN exoskeleton. The primary aim of the study was to assess the stability and ergonomics of the mechanical interaction between the device and the user. Specifically, adopting the comprehensive approach proposed by (D’Elia et al., 2017), we targeted two measurements: (i) the Standard Deviation of the Relative Displacements (RDstd) between the exoskeleton cuffs and the corresponding anatomical part at both thigh and shank level, (ii) the Human-Exoskeleton Kinematic Discrepancy (HEKD) between the exoskeleton kinematics vs. the joint kinematics of the user at both hip and knee level.

Regarding the relative displacements between the exoskeleton cuffs and the corresponding anatomical parts, although some relative displacements are unavoidable due to soft tissue deformability and possible joint axis misalignment, such displacements are expected to remain sufficiently low to avoid excessive shear forces at the contact surfaces. Such forces, in fact, may cause skin irritation or sores and, consequently, discomfort or even dermal tissue wounds (Cempini et al., 2012), especially during prolonged use on fragile people such as those involved in rehabilitation applications.

Another important aspect that is affected by the presence of relative displacement between anatomical and brace segments is the application of stereophotogrammetric 3D gait analysis techniques to people in order to study the functional use of exoskeletons. In fact, given that some anatomical points included in the chosen marker set for gait analysis (Rabuffetti et al., 2019) are hidden by the exoskeleton itself, a typical approach to overcome this problem is to reconstruct the trajectories of the hidden markers through the so called Calibrated Anatomical Systems Technique (CAST), as proposed by (Cappozzo et al., 1995). The CAST approach allows the reconstruction of the position of these markers by using a technical reference frame defined by at least three visible non-aligned markers positioned on the same body segment, or on frames rigidly connected to the body segment, where the hidden marker is located. Using markers placed on the exoskeleton, which are more visible and stable because of the rigid connection among them, enables the definition of a more reliable technical reference frame. Therefore, a reasonable hypothesis is that the exoskeleton segment is rigidly connected to the underlying body segment, ensuring accurate reconstruction of the hidden anatomical marker’s position based on this reference frame. The CAST method relies on the assumption that the anatomical and technical markers are (sufficiently) rigidly connected to each other. Thus, detecting any relative movement between anatomical and exoskeleton segments provides insight into the validity of the above-mentioned rigidity assumption and, as a result, on the reliability of kinematic data derived from 3D gait analysis protocols applied to people while using exoskeletons.

In order to tackle the aforementioned issue, in our study we addressed the measurement of the relative displacement between the exoskeleton cuffs and the corresponding anatomical part using an optoelectronic motion capture system which enabled the recording of both the human and exoskeleton kinematics during walking and STS assisted by the TWIN exoskeleton. The feasibility of measuring displacements on the order of a few millimeters using this technology has already been demonstrated in previous studies (Cempini et al., 2014; D’Elia et al., 2017; Ferrarin et al., 1993).

Regarding the discrepancy between the exoskeleton kinematics vs. the joint kinematics of the user, this estimate represents an important aspect to evaluate, as it describes the ability of the exoskeleton to follow the gait path imposed by the user, and therefore the compatibility between the device and the individual involved (Naf et al., 2018). In addition, this metric may reveal possible joint axis misalignments (D’Elia et al., 2017) which, in turn, can cause residual forces onto anatomical joints and possibly pain or overload-related joint injuries.

The relative displacement and the kinematic discrepancy are expected to be larger during the STS task compared to the walking trial, due to greater joint excursions at the hip and knee level (Cappozzo et al., 1996), which may result in larger relative movements between the orthosis and the underlying anatomy.

## 2 Methods

### 2.1 Participants

Five healthy adults (four female and one male, 60.8 ± 4.8 kg, 1.68 ± 0.02 m, 31.8 ± 5.8 years old) were enrolled for the study. None of them had prior experience with the exoskeleton. All participants signed an informed consent before starting the experimental sessions. The experimental protocol, written in accordance with the Declaration of Helsinki, was approved by the ethical committee of IRCCS Don Carlo Gnocchi Foundation, Milan, Italy (session 21 June 2018). The experiments took place at IRCCS Santa Maria Nascente - Fondazione Don Carlo Gnocchi Onlus (Milan, Italy).

### 2.2 The TWIN exoskeleton

#### 2.2.1 Mechanical structure

The TWIN exoskeleton used for the present research is a powered bilateral lower-limb orthosis developed at the IIT-INAIL Rehab Technologies Lab (Laffranchi et al., 2021). Its original purpose was the rehabilitation of patients with spinal cord injury (SCI), and then it was also employed for post-stroke individuals, thanks to adjustments provided to the control strategy (Semprini et al., 2022).

This orthosis aims to provide tailored support depending on the patient’s residual motor function in order to enhance their learning opportunities and foster neuroplasticity. The main goal is to re-establish a physiological gait pattern, rather than imposing predefined trajectories, thus defining a personalized support approach.

TWIN consists of a pelvis module and two legs, for a total of four structural parts:

- Pelvis module: a “C-Shape” design to ensure comfort. It is available in three sizes to cover the anthropometry measurements (S/M/L). The battery pack and the Central Control Unit (CCU) are located at the pelvis level, on the backside. In particular, the CCU is responsible for the coordination of the actuators, providing measurements and diagnostics (Laffranchi et al., 2021).
- Femur cuff: available in three sizes to cover the anthropometry measurements (S/M/L) (Laffranchi et al., 2021).
- Tibia cuff: also available in three sizes (S/M/L) (Laffranchi et al., 2021).
- Foot: this module, which is located inside the shoe [shoe size: 38 to 46 EU (Laffranchi et al., 2021)], includes a passive elastic ankle joint with a limited rotational mobility.

Each leg is equipped with two actuated joints (motors), located at the hip and knee level, connecting all the parts described above.

**FIGURE 1:**
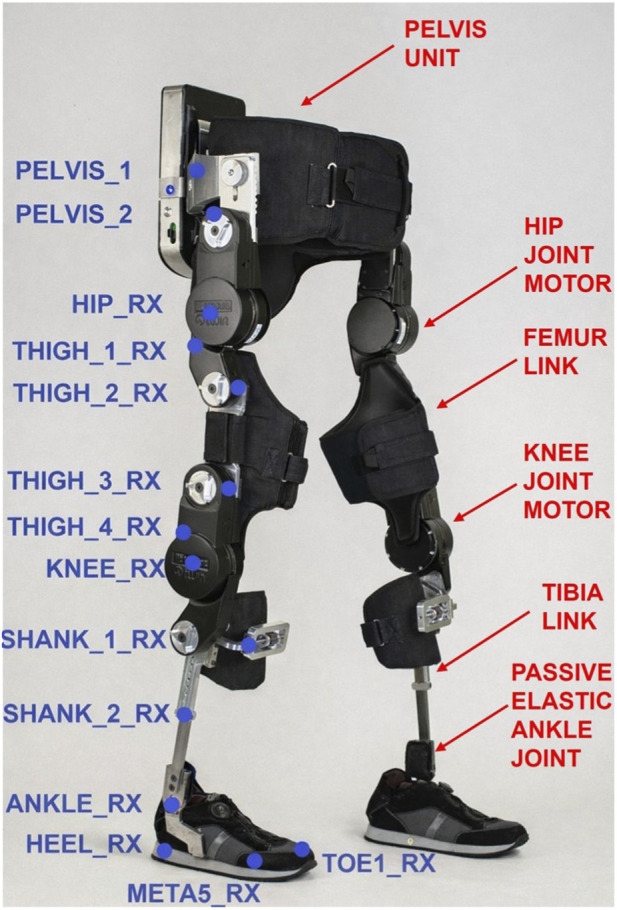
Structure of the TWIN exoskeleton and positioning of “technical” markers (blue points) on the right side of the TWIN exoskeleton during the static calibration and dynamic trials. The markers applied on a single exoskeleton segment (pelvis, thigh, shank, foot) are assumed rigidly connected to each other. The same set of markers is placed on the left side, using the naming convention “LX” instead of “RX”.

The modular structure of the exoskeleton is reported in Figure 1.

Patient and exoskeleton are connected through specific cuffs at the thigh and shank level (for each leg), while a waist cuff is used for the upper part. All cuffs are made of spandex inside that guarantees biocompatibility and low shear forces on the user’s skin, as well as long-term durability, while denim fabric was used for the outer part. They contain Velcro–based straps which help secure the exoskeleton to the patient and minimize relative displacements as much as possible.

The waist cuff consists of a central section connected to the rigid structure through bolts, and two lateral bands that can be fastened around the patient. The frontal part of this cuff helps maintaining a stable posture to ensure balance, preventing potential collapse of the user.

Each thigh allows partial passive rotation around the femur axis, facilitating the donning and doffing of the exoskeleton.

The shank cuff contains a semirigid plate which helps the patient to tilt the tibia in the sagittal plane during the gait.

#### 2.2.2 Control system

TWIN allows to apply two different strategies of walking assistance, based on the residual skills of the user and the level of impairment (Laffranchi et al., 2021).

- Full position control mode: suggested for more severe patients with limited voluntary control, it provides full support during the gait. It is based on the identification of predefined gait trajectories which are computed in the Cartesian space through an interpolation method. It is possible to fully configure the trajectories through parameterization, taking into account step and clearance length (L, H respectively), and step duration (t_s_) for a better stabilization of the walk. In this way, each patient is provided with a reference gait pattern that closely resembles the physiological one, based on the parameters chosen before. Then, the related joint angles are calculated according to the kinematics equations of the system, taking into account the specific exoskeleton dimensions l_H_ (Hip-Centre of Mass distance), l_F_ (Femur link length), and l_T_ (Tibia link length).

In the end, these reference joint positions are sent to the four actuators where are transformed by the local low-level control into a Pulse Width Modulation (PWM) signal that drives the motors.

During the walk, each step is triggered by overcoming two thresholds recorded thanks to the IMU sensor placed in the backpack of the exoskeleton. These two values are obtained based on the angles of trunk inclination (Pitch (P) and Roll (R) angle), defined as the tilt of the pelvis referring to the sagittal and frontal planes, respectively. When P and R exceed the threshold values P_t_ and R_t_Left_ or R_t_Right,_ set according to the patient’s needs, the step trigger is activated.

In addition to trunk inclination, a weight shift from one leg to the other is also required in order to transfer the load onto the supporting leg and allow the swing leg to move forward.

- Assist-as-needed control mode: suggested for patients with residual voluntary motor functions, it helps maximizing learning opportunities. In fact, it is based on a free interaction strategy which allows the user to define the gait trajectory, without imposing predefined paths. Moreover, a certain level of motor assistance is applied in specific gait phases (extension during stance and joint flexion during swing) and it is strictly adapted to the individual’s specific need for support (Semprini et al., 2022). This approach also takes into consideration the non-paretic limb, to which an assistive torque is applied in order to support the weight of the exoskeleton. In this way, a stable posture is ensured, along with the re-establishment of physiological symmetry between the legs in post-stroke individuals, who tend to overuse the healthy leg and avoid shifting weight onto the paretic side. By applying an assistive torque to the impaired limb, the patient is supported in transferring the load onto this leg without causing it to collapse, thereby improving stability and safety while ensuring greater confidence in the hemiplegic limb.

The assist-as-needed strategy is implemented through a control suite named TWIN-Acta. This software is able to identify different gait phases thanks to the four encoders embedded in the exoskeleton hip and knee joints. The information they provide is analyzed by a Finite State Machine (FSM), managed by TWIN-Acta. This classifier runs continuously in the background and determines the state of the active orthosis as well as the phases of the gait cycle based on the kinematic configuration of TWIN.

FSM detects gait phases by computing the inter-feet distance in the sagittal plane, thus only three conditions are possible:

- Right foot forward
- Aligned feet (stance phase)
- Left foot forward

TWIN status depends only on which foot has overtaken the other by a certain distance referred to as the “relative foot distance threshold”. However, this approach does not provide information regarding heel strike events or the shifting of weight from one side to the other. Therefore, a parameter called “transition time” is introduced, which represents the time required for the user to transfer weight from the supporting limb to the opposite one, thus triggering a status change. This time can be configured based on the user’s walking speed.

**FIGURE 2:**
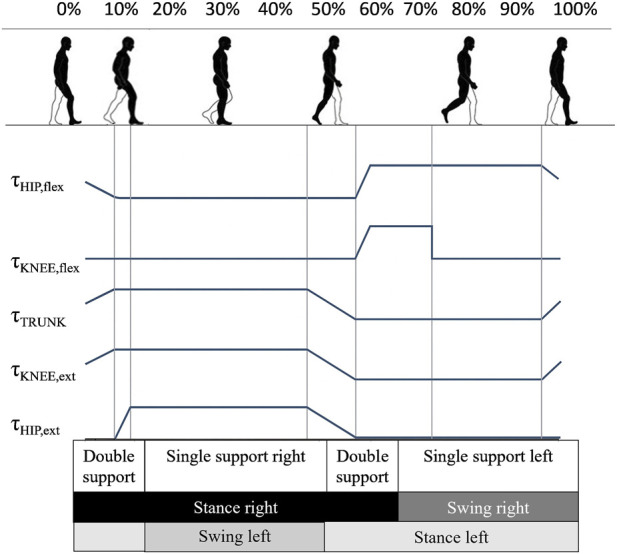
Walking cycle phases and temporal assistance profiles at joints level (modified from (Semprini et al., 2022)).

Finally, the FSM generates control signals to actuate the joints and deliver the necessary assistive torques, according to the impairment of the user. These levels of assistance are not fixed, but they can be adjusted during the experiments in order to provide the most suitable support for both the paretic and non-paretic limbs, compensating also for the friction of the motors and the weight of the exoskeleton (Figure 2). This approach allows torques to be applied only when needed and in different quantities for specific gait phases, based on the motor deficit of each subject.

### 2.3 Experimental protocol

Several calibration and functional acquisition trials were performed on subjects while wearing the exoskeleton.

Firstly, a *Calibration trial (CA)* was acquired to identify all surface markers placed on both the exoskeleton (technical markers, which are supposed rigidly connected) and the human body segments (anatomical markers, of which we want to measure the rigidity of the connection with the technical ones). Moreover, a series of calibration trial were recorded in order to define the positions of some anatomical landmarks not visible to the infrared cameras. These latter ones were collected using a pointer where two markers are mounted on it. Twelve calibration trials were performed. For each one of them, the subject was instructed to remain still in a balanced position for about ten seconds.

Then the following two functional motor tasks were analyzed (dynamic trials):

- *Sit-to-stand (STS):* the subject was asked to rise from a seated position, with both feet flat on the floor, to a standing posture and then to go back to the seated position, using crutches for support. This movement was continuously repeated at least five times per trial, and each acquisition was performed four times per person. Full position control mode was used for the STS task, being the only available control mode of TWIN for this motor task.
- *Walking task (WA):* The subject was asked to walk a short distance (approximately four to five strides) at a steady pace with the support of crutches. This task was repeated at least three times per person. Assist-as-needed control mode, controlled by TWIN-Acta software, was used for the exoskeleton during the walking task, allowing each subject to freely define their own trajectory and the desired assistance level required to ensure stability and symmetry.

### 2.4 Data acquisition and processing

All the experiments took place at “Laboratorio di Analisi del Movimento e Bioingegneria della Riabilitazione (LAMoBiR)” – Fondazione Don Carlo Gnocchi Onlus (Milan, Italy).

Lower limbs kinematic were monitored using an optoelectronic motion capture system equipped with 10 infrared cameras (Smart-DX EVO 9, BTS, Milan, Italy), detecting and recording spherical passive markers placed on specific points on both the subject and the exoskeleton. Smart Tracker Software (BTS, Milan, Italy) was used to obtain the trajectories of the markers during the gait and STS trials.

**FIGURE 3:**
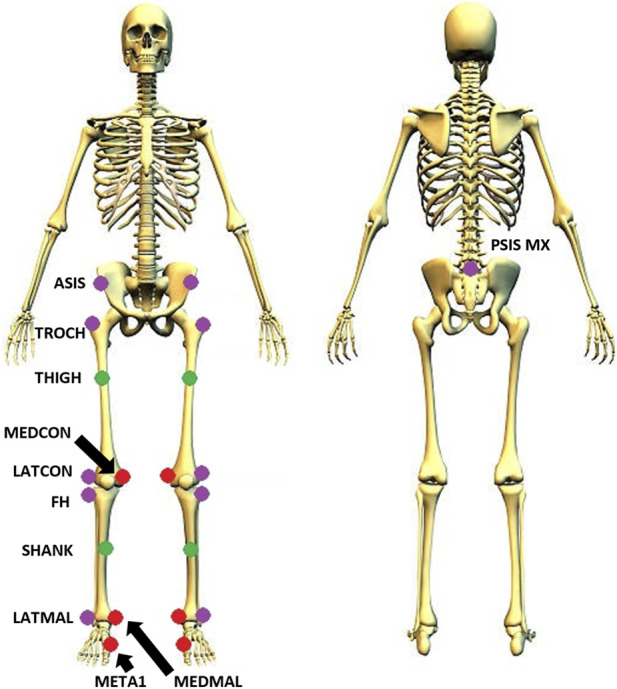
Positioning of markers following the LAMB biomechanical model. Green points: anatomical markers. Red points: calibration markers. Purple points: reconstructed anatomical landmarks identified using a pointer.

Thirty-four markers were positioned on lower-body landmarks and on the TWIN, covering both the lower limbs (Figures 1, 3), following the LAMB model marker setup (Rabuffetti et al., 2019). Twenty-eight of them were placed on the exoskeleton (the blue points in Figure 1) and are called “technical markers” since were used to reconstruct the anatomical landmarks not visible to the cameras of the motion capture system (see CAST procedure described below). Four markers (the green points in Figure 3, called “anatomical segment markers”) were placed on the thigh and the shank of both legs of the participant and were subsequently used to measure the relative displacement between each anatomical segment and the corresponding exoskeleton segment (see Chapter “2.4 Physical human-exoskeleton interface: Relative Displacement (RDstd)”). During the initial calibration, performed through a static trial in which the subject is asked to maintain a steady posture, three additional anatomical markers (identified with the red color in Figure 3) for each leg were added, and removed before the dynamic trials. A second calibration was then carried out using a pointer with two markers mounted on it: this procedure followed the protocol described in (Cappozzo et al., 1995), for those anatomical landmarks hidden by the exoskeleton (purple markers in Figure 3). The calibration process described above is known as Calibrated Anatomical Systems Technique (CAST). The CAST method enables the reconstruction of hidden markers position thanks to the use of a technical reference frame defined by at least three non-aligned markers (visible during the considered motor task) placed on the same body segment, or on frames rigidly connected to the body segment, where the hidden marker is located. Typically, some or even all the three markers defining the technical frame are positioned on the exoskeleton segment, because (1) the latter is more visible than the underlying anatomy and (2) if positioned on the device structural parts, these markers are more rigidly connected to each other, thus making the technical reference frame intrinsically more reliable, under the assumption that the exoskeleton segment is rigidly connected to the underlying body segment. The position of the hidden anatomical landmark is then acquired with respect to the technical frame by means of a calibration static trial, where all markers (both anatomical and technical) are visible and then, during the actual motor task execution, technical and anatomical markers are acquired, while the calibrated ones are not (the red and purple points in Figure 3). Finally, the hidden marker trajectory is reconstructed from the movement of the technical reference frame collected during the motor task execution. As previously stated, a necessary condition for applying the CAST procedure is that the anatomical and technical markers are (sufficiently) rigidly connected to each other, so any relative movement between them can make the procedure unreliable. Thus, the measurement of the relative displacements between anatomical and exoskeleton segments is informative also on the degree of validity of the above-mentioned rigidity assumption and, consequently, on the reliability of kinematic results obtained from 3D gait analysis protocols applied to people while using exoskeletons.

For the purpose of this study, eleven calibration recording were collected (one for each pointed calibration marker), in addition to the first static calibration trial. Since these markers were associated with the pelvis, thigh, and shank segments, the reconstruction of the 3D coordinates did not take the entire foot into account; only the ankle was considered. Data were processed with MATLAB codes (MathWorks, Natick, MA, USA).

For each body segment, three technical markers (placed on the TWIN orthosis) and the point to be reconstructed were considered in order to define a local reference frame and compute the relative coordinates of the calibrated marker. Afterward, the local coordinates were calculated with respect to the global reference system. In this way, the calibrated point was finally reconstructed. This process was applied for the following landmarks, with each landmark identified using the specific cluster of markers:

- Midpoint between the posterior superior iliac spines (PSIS_MX): PELVIS 1 RX, PELVIS 2 RX, PELVIS 2 LX
- Anterior superior iliac spines (ASIS RX/LX): PELVIS 1 RX, PELVIS 2 RX, PELVIS 2 LX
- Greater Trochanters (TROCH RX/LX): THIGH 1 RX, THIGH 2 RX, THIGH 4 RX for the right side; THIGH 1 LX, THIGH 2 LX, THIGH 4 LX for the left side
- Femur lateral condyles (LATCON RX/LX): THIGH 1 RX, THIGH 2 RX, THIGH 4 RX for the right side; THIGH 1 LX, THIGH 2 LX, THIGH 4 LX for the left side
- Head of the fibulas (FH RX/LX): SHANK 1 RX, SHANK 2 RX, ANKLE RX for the right side; SHANK 1 LX, SHANK 2 LX, ANKLE LX for the left side
- Lateral malleoli (LATMAL RX/LX): SHANK 1 RX, SHANK 2 RX, ANKLE RX for the right side; SHANK 1 LX, SHANK 2 LX, ANKLE LX for the left side

We want to underline that the four anatomical segment markers (Figure 3, green points) and the technical markers (Figure 1, blue points) used to estimate the displacement between the exoskeleton cuff and the corresponding anatomical segment, were directly measured by the motion capture system, thus none of them were reconstructed using the CAST procedure.

**FIGURE 4:**
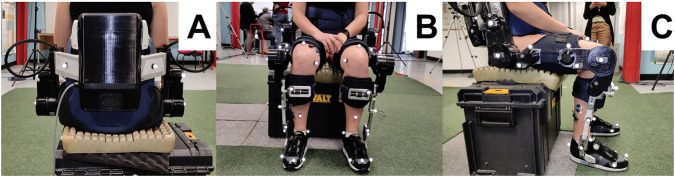
Pictures of a participant showing markers from different views. **(A)** Posterior view. **(B)** Frontal view. **(C)** Lateral view.

The setup is shown in Figure 4.

In order to compare the same trajectories, the encoder data were temporally aligned with those obtained from the Motion Capture system using a peak-based alignment approach. The temporal offset between the two signals was computed and applied to synchronize the recordings, thus enabling the subsequent analyses on the same gait cycle.

Gait cycles (during WA trials) were segmented by selecting two consecutive contact events (heel strike) of the same leg. The heel strikes were identified through the use of three force platforms that detected the ground reaction force during walking. For the STS tests, the initial and final frames of each cycle were identified from the flexion angle of the right hip (it would have been the same using the left hip angle, as the sit-to-stand movement is symmetric across the limbs). In particular, starting from the resting position, the onset of the rising phase was defined as the first frame in which the angle exceeded the resting value by at least 0.5 degrees (for a minimum of ten consecutive frames), whereas the end of the cycle was identified as the minimum between two peaks of the flexion angle, corresponding to a stationary posture of the back, as they represent the sitting phase of the previous cycle and the rising phase of the subsequent one.

Afterwards, all kinematic variables acquired from both the motion capture system and the exoskeleton encoder were time-normalized to 0%–100% of the movement cycle. Finally, stability metrics (see the following sections) were computed for each segmented cycle of both walking and sit-to-stand trials.

### 2.5 Physical human-exoskeleton interface: standard deviation of the relative displacement (RDstd)

From the collected data, the Standard Deviation (SD) of the relative displacements, during the functional trials, between the markers placed on the active orthosis and those placed on their corresponding anatomical segments were computed. Specifically, for the thigh, the SD of the distances between the anatomical marker THIGH and the corresponding technical markers THIGH 1, THIGH 2, THIGH 3 and THIGH 4 were calculated. The individual RDstd index for the thigh was obtained as average of the four previous SD computed values and across all dynamic trial. For the shank, the SD were computed between the anatomical marker SHANK and the corresponding technical markers SHANK 1, SHANK 2 and ANKLE. The individual RDstd index for the shank was obtained as average of the three previous SD calculated data and across all dynamic trials. The same method was applied to both lower limbs, considering the respective markers. The values were computed both for STS and walking trials. The computed parameter, *“Standard deviation of the relative displacement”* (RDstd), represents an index of instability: the larger the value, the more unstable the system, thus leading to greater shifts between the structure and the body segment of the user.

As a reference value, the noise level of the experimental setup was used, computed as the standard deviation of the relative displacement between the technical markers (rigidly connected) calculated across dynamic trials (0.4 ± 0.1 mm).

### 2.6 Human-exoskeleton kinematics discrepancy (HEKD)

A second parameter was calculated from the acquired data, which is the *“Human-exoskeleton kinematics discrepancy”* (HEKD), defined as the Root Mean Square (RMS) of the difference between the flexion/extension (f/e) angle of the exoskeleton joint, recorded by the encoder (having removed the encoder offset at the first sample), and the corresponding anatomical f/e angle, calculated through the motion capture system. This index was estimated at hip and knee joints of both legs. A higher difference may indicate a greater discrepancy between the anatomical joint movement and the corresponding exoskeleton joint one, possibly due to joint axis misalignments.

### 2.7 Statistical analysis

Data normality was verified through the Shapiro-Wilk test. A two-way repeated-measures mixed ANOVA (p < 0.05) was conducted with two within-group factors: joint (two levels: hip and knee) and task (two levels: WA and STS). This analysis evaluates the effects of the joint, task and their interaction on joint kinematic discrepancy. Participants were treated as a random factor. Pre-planned comparisons were performed using the Bonferroni-Holm method, including four comparisons to assess the interaction effect. Statistical analysis was performed using STATISTICA 7.0 (Statsoft, Tulsa, OK, USA).

## 3 Results

All volunteers completed the experimental protocol successfully without reporting discomfort or pain while wearing the TWIN exoskeleton. All recordings were used during the elaboration, except for the STS trials of HS05 and two STS trials of HS03, which were excluded due to data acquisition errors. For each subject, the RDstd and the HEKD were computed individually and then averaged over the group of participants (see “Supplementary Material 1”). Given the absence of differences between the right and the left limb, the results are combined and presented as a single index, pooling data from both legs. The values are expressed in millimeters and degrees, respectively.

### 3.1 Human-exoskeleton interface displacements

**FIGURE 5:**
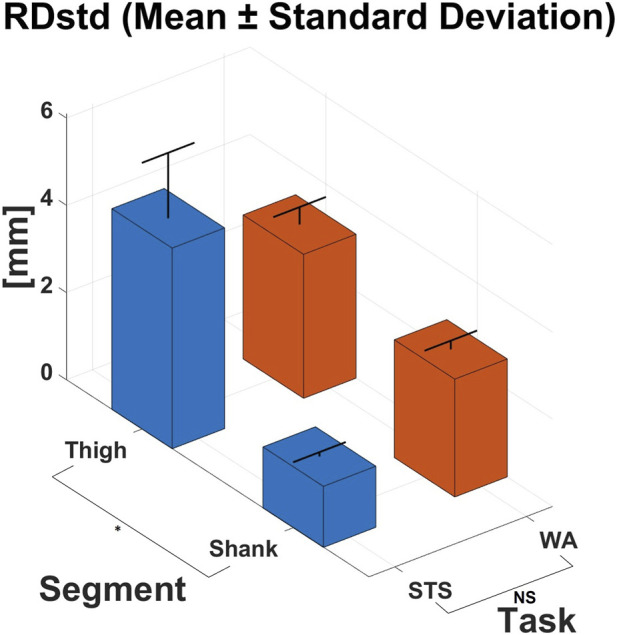
RDstd computed considering an anatomical marker and its corresponding technical markers. The index was computed for each segment (thigh and shank) and task (WA and STS). Values are expressed in mm. *: significant main effect. NS: no significant main effect.

Figure 5 reports the RDstd calculated separately for each task (WA and STS) and segment (thigh and shank), considering a technical marker and its corresponding anatomical one. The reported values are mean ± standard deviation across the subject group.

ANOVA revealed that no significant main effect was present for the type of task performed (p = 0.891, d = 0), but significant effects were found for both the segment (p = 0.007, d = 1.7) and the segment × task interaction (p = 0.007, d = 2.3) across the analyzed trials.

### 3.2 Human-exoskeleton kinematics discrepancy (HEKD)

**FIGURE 6:**
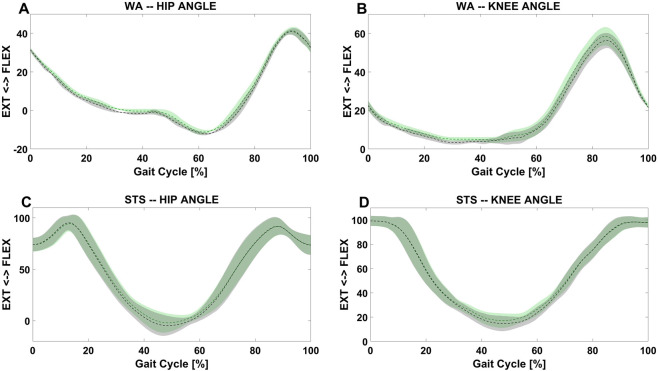
Hip kinematics of the anatomical joint (black dashed lines) and the corresponding exoskeleton joint (green dashed lines) of the right limb, obtained from a representative individual. First row: hip **(A)** and knee **(B)** angle during WA tasks; the line shows the mean stride while the shaded area represents the corresponding SD, both averaged over all trials. Second row: hip **(C)** and knee **(D)** angle during STS tasks; the line shows the mean stride while the shaded area represents the corresponding SD, both averaged over all trials.

Figure 6 presents the hip and knee joint kinematics during both STS and walking trials for a representative subject. The graphs compare the kinematics of the exoskeleton joints with that of the anatomical joints, showing a good agreement in joint angle patterns for both tasks. Larger differences emerge during STS movements, probably due to higher variability.

**FIGURE 7:**
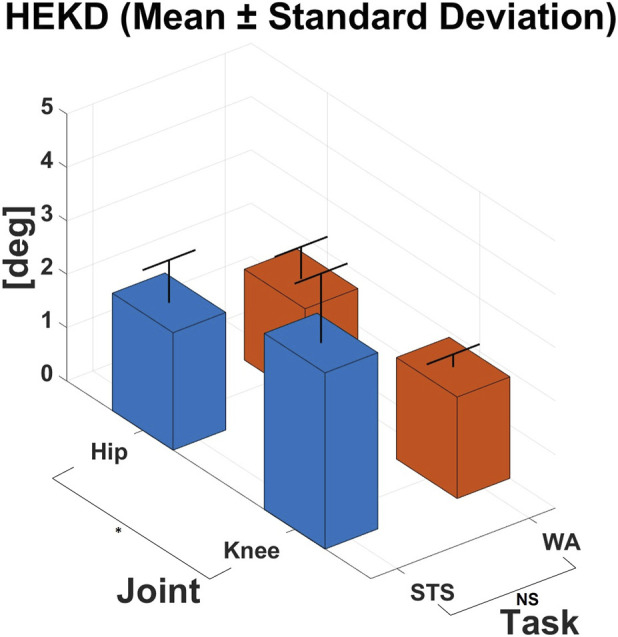
HEKD computed considering the f/e angle, recorded by the joint encoder, and the anatomical f/e angle, calculated through the motion capture system. The index was computed for each task. Values are expressed in degrees. *: significant main effect. NS: no significant main effect.

The HEKD values for each task and joint are reported in Figure 7 (mean ± standard deviation across the subject group).

ANOVA revealed that no significant main effect was present for both the type of task performed (p = 0.276, d = 0.9) and the joint × task interaction (p = 0.107, d = 2.2), but a significant effect was found for the joint (p = 0.047, d = 0.6) across the analysed trials.

## 4 Discussion

Goal of the present study was to assess the stability of the mechanical interaction between the user and the TWIN exoskeleton during functional use and specifically walking and sit-to-stand movements. Two metrics were targeted, according to a previous work carried out by (D’Elia et al., 2017): (i) the relative displacements between the exoskeleton cuffs and the corresponding anatomical part at both thigh and shank level (RDstd), (ii) the discrepancy between the exoskeleton kinematics vs. the joint kinematics of the user at both hip and knee level (HEKD).

### 4.1 TWIN shows reliable mechanical stability during walking

The standard deviation of the relative displacements between the cuffs and the corresponding body segments (RDstd) is considered as a metric of the level of ergonomics assured by the TWIN exoskeleton during sessions of use. High values of RDstd may imply pain or discomfort at the contact points, especially after prolonged use of the device, leading to potential skin wounds.

Even though a total absence of displacements cannot be achieved, it is important to keep these quantities as low as possible.

There is a lack of articles in the literature that establish specific thresholds for the relative displacement between exoskeleton cuffs and the underlying anatomy beyond which discomfort or skin damage may occur. To the best of our knowledge, (Mahmud et al., 2010), is the only study providing quantitative data relevant to this issue. They observed no skin damage or pain for skin strains of up to 11.7 mm, a value significantly larger than the cuff-anatomy relative displacements measured in the present study. This comparison supports the interpretation that the displacements observed here are unlikely to be critical in terms of pain or risk of skin injury.

Interestingly, differences in RDstd metric were observed both between motor tasks and across anatomical segments. Specifically, RDstd values at the shank were slightly lower than those at the thigh for both motor tasks (WA: RDstd_thigh_ = 3.3 ± 0.4 mm, RDstd_shank_ = 2.7 ± 0.2 mm; STS: RDstd_thigh_ = 4.6 ± 1.5 mm, RDstd_shank_ = 1.4 ± 0.1 mm). This difference may have two main explanations. First, it could be attributed to the greater structural constraint of the shank, partly due to the presence of the anterior metal plate (Figure 1), which limits the relative movements between the limb and the orthosis. Conversely, the thigh brace allows for greater relative movements, including not only sliding but also rotational displacements around the femoral axis. This effect becomes particularly evident during the STS task. Second, the anatomical thigh marker is placed over the quadriceps, a bulky muscle that can move with respect to the thigh brace during contraction; in addition, skin elasticity and soft tissue compliance in this region may further amplify the observed displacement. In contrast, the anatomical shank marker is placed on the medial/anterior region of the shank, where subcutaneous tissues are very thin, thus limiting possible spurious relative movements between the skin and the underlying bone (i.e. the “skin movement artefact” commonly referenced in marker-based motion capture). As previously stated, according to (Cappozzo et al., 1996), increased joints movement leads to greater artefacts in identifying anatomical landmarks; therefore, any shift in marker position affects the application of a 3D gait analysis protocol. This effect is amplified at the thigh, as this segment is larger than the shank and thus makes the computation of thigh kinematics more sensitive to inaccuracies in the definition of its reference system. Due to the limited sample size, this observation should be interpreted as a trend rather than a definitive effect, as discussed earlier.

As expected, there was a tendency toward larger relative movement between the thigh cuff and its corresponding anatomical segment during STS compared to walking (WA: RDstd_thigh_ = 3.3 ± 0.4 mm; STS: RDstd_thigh_ = 4.6 ± 1.5 mm). This is likely due to the greater hip and knee joint excursions involved in STS task, which are substantially larger than those occurring during level walking and are known to amplify soft-tissue displacements and skin–brace relative motion (Cappozzo et al., 1996). Interestingly, this phenomenon was not observed at the shank, where slightly higher RDstd values were recorded during walking than during STS (WA: RDstd_shank_ = 2.7 ± 0.2 mm; STS: RDstd_shank_ = 1.4 ± 0.1 mm). A plausible explanation is that foot-ground impacts during walking introduce destabilizing effects on the lower leg, promoting additional movements between the segment and the cuff. Such effects are not present during STS, a task in which the shank remains nearly stationary, partly due to the very limited motion allowed by the TWIN at the ankle joint.

Compared to results reported in the reference study by (D’Elia et al., 2017), which showed RDstd values at the thigh between 4.9 and 6.3 mm during walking tasks with a hip-active orthosis, TWIN exhibited lower values (3.3 mm), indicating greater mechanical stability at this segment. With respect to the reference study, all the considerations for RDstd regarded only the thigh segment, as the pelvis was fully enclosed, in our study, by the TWIN structure due to the presence of a large pelvis cuff, which prevented the placement of anatomical markers directly on the pelvis. Although such a design could plausibly enhance mechanical stability by providing a broader and more rigid fixation at the pelvic level, further analyses will be required to confirm this hypothesis.

### 4.2 TWIN ensures stable coupling with the subject during dynamic tasks

The RMS of the difference between the joint angle pattern of the TWIN exoskeleton joint and the corresponding anatomical joint angle pattern - referred to as the Human-Exoskeleton Kinematic Discrepancy (HEKD) - during a functional movement can considered an indicator of the user-device coupling quality, particularly during dynamic tasks such as those analyzed in this study. Low HEKD values are desirable, as they indicate that the exoskeleton joint motion closely follows the subject’s actual anatomical motion, thereby suggesting good compliance between the user’s anatomy and the exoskeleton. The comparison of the HEKD results obtained in the present study with those reported by (D’Elia et al., 2017) was limited to the hip joint, since the device employed in their work featured only an actuated hip joint and not a knee joint.

As reported in Figure 7, HEKD values for the hip are 1.7 ± 0.6 deg for WA and 2.2 ± 0.8 deg for STS. For the knee, HEKD ranges between 1.9 ± 0.4 and 3.3 ± 1.3 deg for WA and STS, respectively. With respect to (D’Elia et al., 2017), who found HEKD values at the hip joint spanning from 2.7 to 7.2 degrees (based on the selected walking modality), our results at the hip joint are slightly lower.

A tendency toward larger discrepancies is observed during STS compared to walking for both hip and knee joints. Consistent with what was previously noted for the RDstd index, this may be attributed to the greater joint excursions involved in STS movements relative to those required during walking (Cappozzo et al., 1996). Additionally, for both tasks, HEKD_hip_ is lower than HEKD_knee_, indicating a better coupling between the TWIN exoskeleton and the user at the hip level than at the knee level. This is likely related to the mechanical configuration of the exoskeleton: the rigid pelvic frame is expected to provide a more stable interaction with the user’s pelvis, effectively constraining hip motion and thereby reducing the kinematic discrepancy, as already noted. Moreover, these results may be further explained by the shift in the knee instantaneous center of rotation during flexion/extension movements, in contrast to the hip joint, which maintains a fixed center of rotation due to its ball-and-socket joint structure. However, although the effect sizes were large, the ANOVA test did not reveal statistically significant differences with respect to the type of trial or the joint × task interaction, suggesting that these findings should be interpreted as a tendency rather than a definitive effect.

### 4.3 A framework for evaluating and quantifying ergonomics and its implications in the present use-case

Overall, considering parameters such as RDstd alongside HEKD provides valuable insights into the mechanical interaction between the subject and the powered lower-limb device, with possible consequences in terms of development of skin injuries and pain onset. Human-exoskeleton kinematic configuration can be characterized through these two indices, as low values of RDstd and HEKD imply minimal joint axis misalignment, while high values suggest significant joint misalignment and/or looseness between cuffs and body segments. In both cases, sliding relative movements can occur, which may result in discomfort and potential skin wounds. These aspects are particularly critical in exoskeleton-assisted rehabilitation, where suboptimal human–robot coupling may not only reduce the effectiveness of the treatment but also risk fostering maladaptive behaviors. Moreover, a robust mechanical interaction between individual and device allows for monitoring user’s movements without needing extra markers, speeding up rehabilitation sessions. Ultimately, these insights offer valuable guidance for improving exoskeleton design.

In our study, RDstd values for the two considered motor tasks at both thigh and shank level were found significantly lower than 11.7 mm, a safety value for the possible onset of discomfort and skin irritation, according to the experimental results of (Mahmud et al., 2010). HEKD data are not critical, as the difference between the two angles is only about 2 degrees for the hip and 3 degrees for the knee (in the worst-case scenarios). None of the participants reported pain or the presence of skin lesions after the tests. Therefore, it is possible to affirm that the TWIN exoskeleton does not cause discomfort or risk of any kind of injury, despite the presence of some certain displacement between the cuffs and the corresponding body parts.

The proposed metrics requires further investigation in order to develop a robust framework for evaluating and quantifying ergonomics, also in relation to various conditions of use of the exoskeleton (e.g. different walking speeds, variable assistance levels, additional locomotor tasks). Moreover, it could be useful to further investigate the human-exoskeleton interaction by estimating the interaction forces at the cuff level (Torricelli et al., 2020), as, if too high, may lead to unwanted effects such as skin injuries (Massardi et al., 2023b). Quantifying the shear and compressive components of these forces could help design more ergonomic and comfortable braces (Langlois et al., 2018), minimizing undesired parasitic loads on the user’s musculoskeletal system and ensuring a broader pressure distribution area. Furthermore, analyzing the forces at the interaction surfaces would provide insights into the quality of the assistance delivered by the actuated device.

Our findings, despite being based solely on sit-to-stand and walking tasks and with a cohort of healthy volunteers, indicate that TWIN holds promise for use in neurorehabilitation. In future studies, by incorporating a wider variety of tasks into the training session, we will better assess the device mechanical stability to validate its suitability for gait rehabilitation.

### 4.4 Limitations of the study

This study has some limitations.

First, the sample consisted of only five healthy participants, reflecting the pilot nature of the investigation. Although traditional assessments of human–machine interface ergonomics typically require larger cohorts due to reliance on subjective measures such as usability and comfort questionnaires, the present study employed an objective, motion capture–based methodology capable of detecting minimal relative displacements between the exoskeleton and the user’s body. Nevertheless, the small sample size reduces the generalizability of the findings and limits the ability to capture inter individual variability. Furthermore, previous studies using similar objective approaches have also relied on small cohorts (e.g., (Cempini et al., 2014; D’Elia et al., 2017)), which, while supporting methodological comparability, underscores the need for future research involving larger and more heterogeneous populations.

Second, the present work focused on short-term testing and did not address long-term durability or performance over extended use. Future studies will therefore need to include longer follow-up periods and larger clinical trials to fully assess durability, reliability, and performance in real-world rehabilitation settings.

A final limitation of this study is the lack of direct measurements of interaction forces between the exoskeleton and the user. Such data would be valuable for a more comprehensive assessment of comfort, safety, and human–robot interaction during use. However, the current version of the exoskeleton is not equipped with force sensors at the joints, which prevented the acquisition of these measurements. Future iterations of the device will aim to integrate force-sensing capabilities to enable more detailed analyses of user–device interaction and further support ergonomic and safety evaluations.

## 5 Conclusion

In this paper, the interface between a powered-lower-limb exoskeleton, TWIN, and the user was investigated in order to assess the ergonomics of the device, focusing on the potential onset of pain, discomfort, or risk of skin injuries.

To this end, the standard deviation of the relative displacement (RDstd) and the root mean square of the difference between the TWIN joint and the anatomical joint angles (HEKD) were calculated during walking and Sit-to-Stand tasks executed by five healthy volunteers with the assistance of TWIN.

Compared to data reported in literature, both indices showed consistently low values across tasks and segments/joints, hence there was no significant relative displacement between the cuffs and the underlying anatomical segments and kinematic discrepancies can be interpreted as non-critical. Moreover, none of the participants reported any discomfort or pain, suggesting that the device provides both stability and support during the sessions.

With respect to the initial hypothesis, instability and kinematic discrepancy were generally greater during STS compared to WA. Only RDstd_shank_ was lower in STS than WA, probably due to the destabilizing impact of the foot to the ground during walking, as previously discussed. These findings will inform future design revisions of the device, such as the optimization of the cuffs, to minimize the observed discrepancies and further improve user interaction.

For now, RDstd and HEKD can be considered valid indicators of comfort provided by TWIN, given the observed correlation between the measured displacement, the kinematic discrepancy and the risk of injury.

Future steps of the present study include the enrollment of participants with different anthropometric characteristics and clinical conditions, in order to strengthen the external validity and fully assess the robustness and generalizability of the obtained results. In particular, the long-term use of the TWIN exoskeleton will be investigated to: (i) monitor the occurrence of skin injuries, (ii) assess the perception of discomfort in target clinical populations (e.g. post-stroke and spinal cord injured patients), and (iii) analyse the relationship between these potential issues and the proposed metrics.

## Data Availability

The raw data supporting the conclusions of this article will be made available by the authors, without undue reservation.

## Supplementary material

SUPPLEMENTARY TABLE 1 RDstd and HEKD individual values and ANOVA results.
